# Extracellular Superoxide Dismutase Regulates the Expression of Small GTPase Regulatory Proteins GEFs, GAPs, and GDI

**DOI:** 10.1371/journal.pone.0121441

**Published:** 2015-03-09

**Authors:** Mikko O. Laukkanen, Francesca Cammarota, Tiziana Esposito, Marco Salvatore, Maria D. Castellone

**Affiliations:** 1 IRCCS SDN, Naples, Italy; 2 Department of Biomorphological and Functional Sciences, University of Naples Federico II, Naples, Italy; 3 Institute of Experimental Endocrinology and Oncology (IEOS/CNR), Naples, Italy; 4 Department of Molecular Medicine and Medical Biotechnologies, University of Naples Federico II, Naples, Italy; IBMC - Institute for Molecular and Cell Biology, PORTUGAL

## Abstract

Extracellular superoxide dismutase (SOD3), which catalyzes the dismutation of superoxide anions to hydrogen peroxide at the cell membranes, regulates the cellular growth in a dose-dependent manner. This enzyme induces primary cell proliferation and immortalization at low expression levels whereas it activates cancer barrier signaling through the p53-p21 pathway at high expression levels, causing growth arrest, senescence, and apoptosis. Because previous reports suggested that the SOD3–induced reduction in the rates of cellular growth and migration also occurred in the absence of functional p53 signaling, in the current study we investigated the SOD3-induced growth-suppressive mechanisms in anaplastic thyroid cancer cells. Based on our data, the robust over-expression of SOD3 increased the level of phosphorylation of the EGFR, ERBB2, RYK, ALK, FLT3, and EPHA10 receptor tyrosine kinases with the consequent downstream activation of the SRC, FYN, YES, HCK, and LYN kinases. However, pull-down experiments focusing on the small GTPase RAS, RAC, CDC42, and RHO revealed a reduced level of growth and migration signal transduction, such as the lack of stimulation of the mitogen pathway, in the SOD3 over-expressing cells, which was confirmed by MEK1/2 and ERK1/2 Western blotting analysis. Interestingly, the mRNA expression analyses indicated that SOD3 regulated the expression of guanine nucleotide-exchange factors (*RHO GEF16*, *RAL GEF RGL1*), GTPase-activating proteins (*ARFGAP ADAP2*, *RAS GAP RASAL1*, *RGS4*), and a Rho guanine nucleotide-disassociation inhibitor (*RHO GDI 2*) in a dose dependent manner, thus controlling signaling through the small G protein GTPases. Therefore, our current data may suggest the occurrence of dose-dependent SOD3–driven control of the GTP loading of small G proteins indicating a novel growth regulatory mechanism of this enzyme.

## Introduction

Cell membrane-bound extracellular superoxide dismutase (SOD3) is one of the three SOD isoenzymes that catalyze the dismutation of superoxide radical (O_2_^.-^) to hydrogen peroxide (H_2_O_2_) [1]. Notably, both O_2_^.-^ and H_2_O_2_ are second messengers in cell signaling [2,3] suggesting that the cellular effects of SOD3 are mediated by the local reduction of the O_2_^.-^ concentration and the simultaneous increase of the H_2_O_2_ concentration affecting the activation of the cell membrane-bound receptor tyrosine kinases (RTK), with a consequent impact on downstream growth and migration signaling [4,5,6]. Consistent with this hypothesis, several studies have demonstrated that SOD3 regulated growth and survival related signal transduction and gene expression [5,6,7,8,9,10,11], hence emphasizing the importance of this enzyme in growth regulation. Interestingly, *SOD3* expression is mildly upregulated in benign tumor model systems whereas it is downregulated in several cancers and in transformed cell lines [12,13] suggesting that the enzyme might be involved in the initiation of benign hyperplasia.

Based on our recent data SOD3 has a dose-dependent effect on cellular proliferation; low expression levels of SOD3 induce GTP loading on small GTPase proteins, mitogen signaling and cell proliferation, whereas high levels of SOD3 activate the DNA-damage response and the p53-p21 growth arrest pathway [5,6]. Although activation of the p53-p21 signal transduction pathway is likely to play a major role in growth limitation, high-dose SOD3–inhibited proliferation was also observed in anaplastic thyroid cancer cells lacking functional p53 [5], indicating the existence of additional growth regulatory mechanisms.

Therefore, in the current study we focused on examining the effect of high-dose SOD3 on cell membrane-associated growth-regulatory molecules to identify the target molecules that translate the enzyme-based signaling to the cellular signal transduction network. According to our current data high-level SOD3 expression activated growth signaling through the cell membrane-bound receptor tyrosine kinases (RTKs) and cytoplasmic non-receptor tyrosine kinases (TKs). However, simultaneously with the increased phosphorylation of RTKs and TKs, high-dose SOD3 decreased the level of expression of a number of growth-promoting genes, downregulated the activation of the RAS, RHO, RAC, and CDC42 small GTPases, and controlled the expression of the small GTPase regulatory genes encoding guanine nucleotide-exchange factors (GEFs), GTPase-activating protein (GAPs) and Rho guanine nucleotide dissociation inhibitor (GDI). These results could therefore clarify how SOD3 controls cellular proliferation and may additionally suggest potential drug targets for reducing carcinogenic growth.

## Methods

### Cell lines

8505c cells (DSMZ, German collection of microorganism and cell cultures, Braunschweig, Germany) modeling anaplastic thyroid cancer, were cultured in RPMI medium (Sigma, St. Louis, MO, USA) supplemented with 10% FBS. Cell lines stably expressing human *SOD3* (kindly provided by Professor Stefan L. Marklund of the University of Umeå, Sweden), human *RHO GEF16* (Applied Biological Materials, Richmond, Canada), *H-RasV12* or the pcDNA3 *GFP* control plasmid were utilized. Cell lines were generated by nucleofection of 5 μg of the *SOD3* expression plasmid or the control plasmid into 5x10^5^ cells, 5 μg of *SOD3*/1.5 μg of *H-RasV12*, and 5 μg of *SOD3*/1.5 μg of *RHO GEF16* transfection into 5x10^5^ cells. The appropriate antibiotic selection was applied 48 hours after transfection and was continued for six weeks to create stable mixed cell populations. N-acetyl-cysteine (NAC) (Sigma) was applied to the 8505c cells at 2.5 mM daily.

### Growth analysis

For the growth curve analyses 5x10^3^ cells were seeded in the wells of 6-well plates in triplicate, and were counted daily until the cells reached a maximum of 70% confluence to avoid artifacts caused by cellular overgrowth. For the BrdU cell proliferation cell proliferation analyses, 10 mM bromodeoxyuridine (BrdU) (Roche, Basel, Switzerland) was added to the growth medium for 15 min. Subsequently, the cells were fixed using an ethanol fix solution. The BrdU-positive cells were detected using FITC–conjugated secondary antibodies (Jackson ImmunoResearch Laboratories Inc., West Grove, PA). The nuclei were counter stained using Hoechst (Sigma) staining. Each cell count was performed in triplicate: each point represents the mean value for 3 samples.

### Invasive growth in Matrigel

For outgrowth in Matrigel, 1x10^3^ stably transfected SOD3 and control plasmid were mixed with 160 μl of Matrigel (BD Biosciences, San Jose, CA, USA) and were plated in 35 mm dishes containing glass coverslips. After the gel hardened, the cells were overlaid with 2 ml of growth medium and were incubated at 37°C for up to 8 days.

### Soft agar assay

*GFP* control and *SOD3* transfected 8505c cells (5x10^3^) were plated in 6 cm gridded dishes with a bottom layer of 1% agarose and a top layer of 0.4% agarose constructed using complete growth medium. The plates were refreshed with complete medium twice a week. The number of colonies in each grid was counted at the 6-week time point. Each count was performed in triplicate.

### Protein arrays

Proteins were isolated from 8505c cells stably expressing *SOD3* or *GFP* control. Human phospho-RTK and phospho-kinase arrays (R&D Systems, Minneapolis, MN USA) were performed using 300 μg of protein according to the manufacturer’s instructions. Signal density analysis was performed using ImageJ software GEL blot software.

### Pull-down analysis

RAS, RAC, CDC42, and RHO pull-down analysis was performed using cells at 40% confluence in 10 cm dishes. After serum starvation for 24 h, the cells were lysed using ice-cold Rho-lysis buffer containing 20 mM HEPES (pH 7.4), 0.1 M NaCl, 1% Triton X-100, 10 mM EGTA, 40 mM glycerophosphate, 20 mM MgCl2, 1 mM Na3VO4, 1 mM dithiothreitol, a mixture of protease inhibitors, and 1 mM phenylmethylsulfonyl fluoride. The lysates were incubated for 15 min with a purified, bacterially expressed GST-fusion protein containing the CRIB domain of PAK1 (p21 activated kinase) that had been previously bound to glutathione-Sepharose beads, followed by three washes using Rho-lysis buffer. The GTP-bound forms of RAC1 or CDC42 associated with GST-CRIB were quantified through Western blotting analysis. GST-Rhotekin-RBD and GST-RAF1-RBD beads were used for RHO and RAS pull-down assay, respectively, and analyzed by Western blotting.

### Western blotting analysis

The cells were homogenized in lysis buffer (50 mM/L HEPES pH 7.5, 150 mM/L NaCl, 10% glycerol, 1% Triton X-100, 1 mM/L EGTA, 1.5 mM/L MgCl_2_, 10 mM/L NaF, 10 mM/L sodium pyrophosphate, 1mM/L Na_3_VO_4_, 10 μg approtinin/ml, 10 μg leupeptin/ml) (Sigma). The following antibodies were used: α-RAS, α-RAC, α-CDC42, α-RHO, α-pEGFR, α-EGFR, α-pFAK, α-FAK, α-pSCR, α-SCR, α-pMEK, α-MEK, α-pERK1/2, α-ERK1/2, α-pAKT, α-AKT, α-pGSK3 α /β, α-GSK3 α /β, α-β catenin, α-pCHK2, α-CHK2, α-tubulin, α-SP1, α-pRET, α-RET (Cell Signaling, Danvers, MA, USA), and α-SOD3 (Santa Cruz, Santa Cruz, CA, USA). Signal density analysis was performed using ImageJ software GEL blot software.

### DNA damage assays

For γH2AX staining (Millipore, Billerica, MA, USA) the cells were grown on coverslips, fixed using 4% PFA/PBS for 20 min at room temperature, permeabilized using 0.5% TritonX-100 and incubated with a primary antibody directed against γH2AX for 1h at RT. After several washes and labeling using the secondary antibody (30 min at RT), the cells were counterstained using Hoechst dye and were analyzed using fluorescence microscopy. Cells containing more than ten nuclear foci were considered positive.

### Microarray

For the microarray expression analysis, the RNA was extracted from the 8505c cells in triplicate for use in cDNA synthesis. The analysis was performed at the Finnish Microarray and Sequencing Centre (FMSC), Turku Centre for Biotechnology, University of Turku, Finland. In brief, the array analysis was performed using an Illumina Human HT-12 v.3 Expression BeadChip that contained more than 48 000 types of probes according to the instructions of the manufacturer. Washing and scanning were performed according to the instructions in the Illumina BeadStation 500x manual (revision C). The Illumina expression data were extracted using BeadStudio version 1.5.0.34 software applying the default settings. The following quality assessments were applied to the samples: RNA quality control, quintile normalization of the sample data, inspection of the signal-intensity distribution, and sample correlation analysis. The threshold values for positive hits were selected based on the MA plot, and volcano blot comparisons were used to identify list the most differentially expressed array features. Three functional pathway analytical tools, the KEGG pathway signaling (http://www.genome.jp/kegg/pathway.html), GOrilla GO signaling pathway (http://www.ebi.ac.uk/QuickGO/), and GSEA gene enrichment programs were used to connect the gene expression patterns to cellular functions. The statistical analysis was performed using the R/Bioconductor6 open software package. The resulting gene list was filtered according to P G 0.001 (without adjustment for multiple testing). The genes were annotated, and output files were created using the biomaRt package. The selected positive hits were double-checked using quantitative RT-PCR.

### Gene expression analysis using real-time RT-PCR

The total RNA was isolated from the cells using an RNeasy minikit (Qiagen, Hilden, Germany). The first strand synthesis was performed using a QuantiTect Reverse Transcription kit (Qiagen) and quantitative PCR was performed using the SYBR Green PCR master mix (Applied Biosystems, Foster City, CA, USA). The primers are listed in S1 Table.

### Analysis of reactive oxygen species

Human 8505c control, 8505c SOD3, 8505c SOD3 Ras, and 8505c SOD3 GEF16 cells were grown on coverslips. Dihydroethidium (Sigma) and MitoTracker RED CMX (Invitrogen, Waltham, MA, USA) were added to the cells in 100 nM final concentrations. Cells were incubated 45 minutes at 37°C and washed with PBS. Images were analyzed with fluorescence microscope.

### Mouse transplantation

Human 8505c cells (10x10^6^ cells) that were stably transfected with *luciferase* or *SOD3* were transplanted subcutaneously into two locations of the dorsal portion of female BALB/c nude (nu/nu) mice. The tumor size was assessed using a caliper at regular intervals and the tumor volume was calculated according to the following formula: (*LxW* ^*2*^ *)/2* (where L = length and W = width of tumor). The mice were sacrificed four weeks after transplantation and the tumors were isolated for RNA preparation (5). All of the animal procedures were approved by the Southern Finland Regional Experimental Animal Committee (License STH349A), and were performed according to the European Commission guidelines.

### Statistical analyses

The experiments were repeated at least three times. All of the results were expressed as the mean values ±SD. The p-values (* = p<0.05, ** = p<0.01, *** = p<0.001) were determined using a one-way ANOVA with a Tukey-Kramer multiple comparison post-analysis test or, when appropriate, using two-tailed independent sample t-tests.

## Results

### Robust *SOD3* over-expression reduced the rates of anaplastic thyroid cancer cell proliferation, invasion, and anchorage independent growth *in vitro*

We previously showed that robust SOD3 over-expression reduced the rate of cell proliferation by causing DNA damage, inducing the DNA-damage response, and activating the downstream p53-p21 signal transduction pathway of TPC1 papillary thyroid cancer cells, which have an intact growth-arrest signaling pathway. Notably, robust SOD3 over-expression also reduced the rate of cell proliferation of anaplastic thyroid cancer 8505c cells, which lack functional growth-arrest signaling [5]. Therefore, in the current study, we sought novel SOD3–related signal transduction routes and the molecules that could transmit the SOD3-based extracellular signals to the cytoplasmic signaling network.

**Fig 1:**
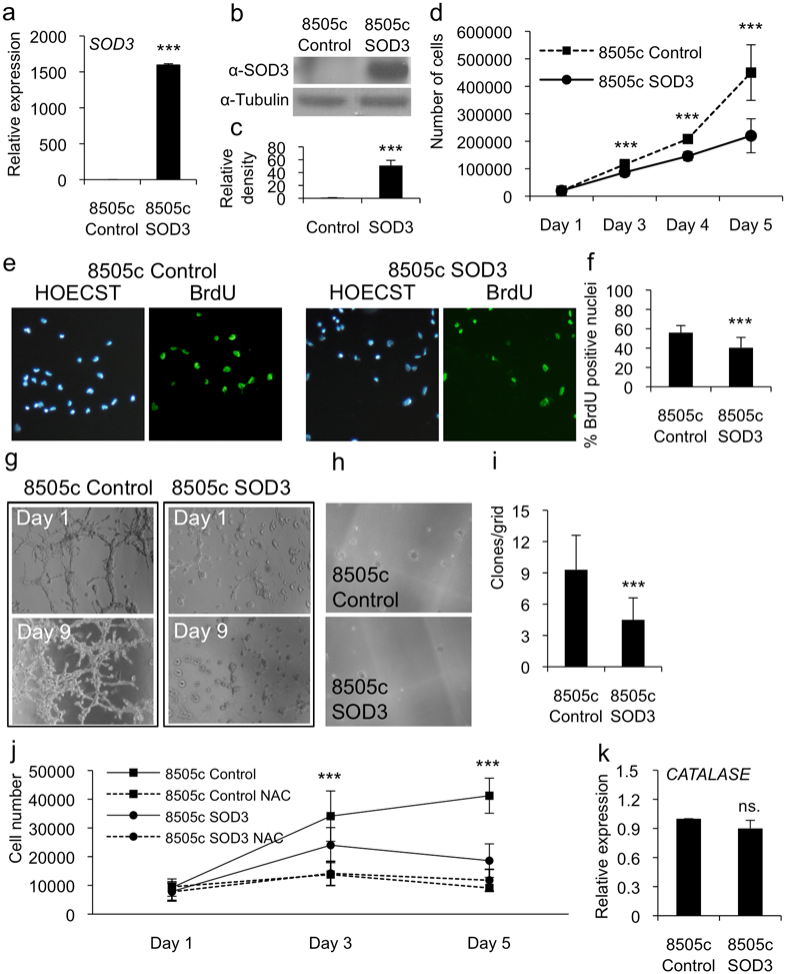
Robust SOD3 expression reduced anaplastic thyroid cancer cell growth. (a) Real time RT-PCR analysis showed a high-level mRNA production of *SOD3* transgene in anaplastic thyroid cancer 8505c cells. (b,c) Western blotting and related band intensity analysis showed high-level SOD3 production caused by *SOD3* transgene. (d) The growth curve analysis suggested significantly (p<0.001) decreased cell numbers in cells transfected with *SOD3*. (e,f) The BrdU DNA incorporation analysis confirmed significantly (p<0.001) decreased cellular growth caused by SOD3. (g) The 3-D gel invasion assay suggested almost complete lack of invasive capacity of 8505c SOD3 cells. (h,i) The soft agar analysis showed significantly (p<0.001) reduced formation of colonies suggesting decreased anchorage independent growth ability for SOD3 cells as compared to 8505c control vector transfected cells. (j) The growth curve analysis of control and SOD3 cells in the presence of 2.5 mM N-Acetyl-cysteine (NAC) showed growth reduction both in 8505c control and SOD3 expressing cells upon the treatment. (k) *CATALASE* mRNA expression. The quantitative RT-PCR showed no difference between 8505c control and SOD3 cells in *CATALASE* expression. Data are expressed as mean ±SD. The p-values (* = p<0.05, ** = p<0.01, *** = p<0.001).

To evaluate the level of SOD3 synthesis, we determined the expression level of transfected *SOD3* using real-time RT-PCR and Western blotting which demonstrated high-level mRNA and protein production in anaplastic 8505c cells (Fig. 1a-1c). To evaluate the effect of SOD3 over-expression on biological processes we studied the effect of SOD3 on the aggressiveness of these cancer cells. The growth curve analysis of anaplastic 8505c cells robustly over-expressing SOD3 demonstrated significantly (p<0.001) reduced cell numbers (Fig. 1d) and BrdU positive proliferating nuclei (Fig. 1e and 1f) compared with those of the control cells, consistent with our previous data [5]. Furthermore, the SOD3 over-expressing cells showed a reduced level of invasive growth in Matrigel (Fig. 1g) and colony formation in soft agar (p<0.001) as compared with those of the control cells (Fig. 1h and 1i). The data therefore suggested a reduced aggressiveness of the anaplastic thyroid cancer cells caused by high SOD3 expression.

Because SOD3 regulates the availability of superoxide anion and hydrogen peroxide reactive oxygen species, we tested the effect of the general ROS scavenger N-acetyl-cysteine (NAC), which mainly removes superoxide but can also reduce the H_2_O_2_ and nitric oxide (NO) concentrations, on cell proliferation. Noteworthy, O_2_^-^, H_2_O_2_, and NO derivative peroxynitrate can increase growth by oxidative modification of MEK1/2-ERK1/2 signaling [2,3,14]. The growth curve analytical data showed reduced numbers of 8505c control cells in the presence of 2.5 mM NAC compared with those of the cells grown without NAC. This treatment even decreased the rate of proliferation of 8505c SOD3 over-expressing cells compared with that of untreated 8505c SOD3 cells, emphasizing the role of oxygen and nitrogen free radicals in this process (Fig. 1j). Interestingly, SOD3 over-expression had no impact on catalase expression (Fig. 1k) suggesting differential signal transduction for the enzymes.

### SOD3 affected the activation of the cell membrane-associated receptor tyrosine kinases

**Fig 2:**
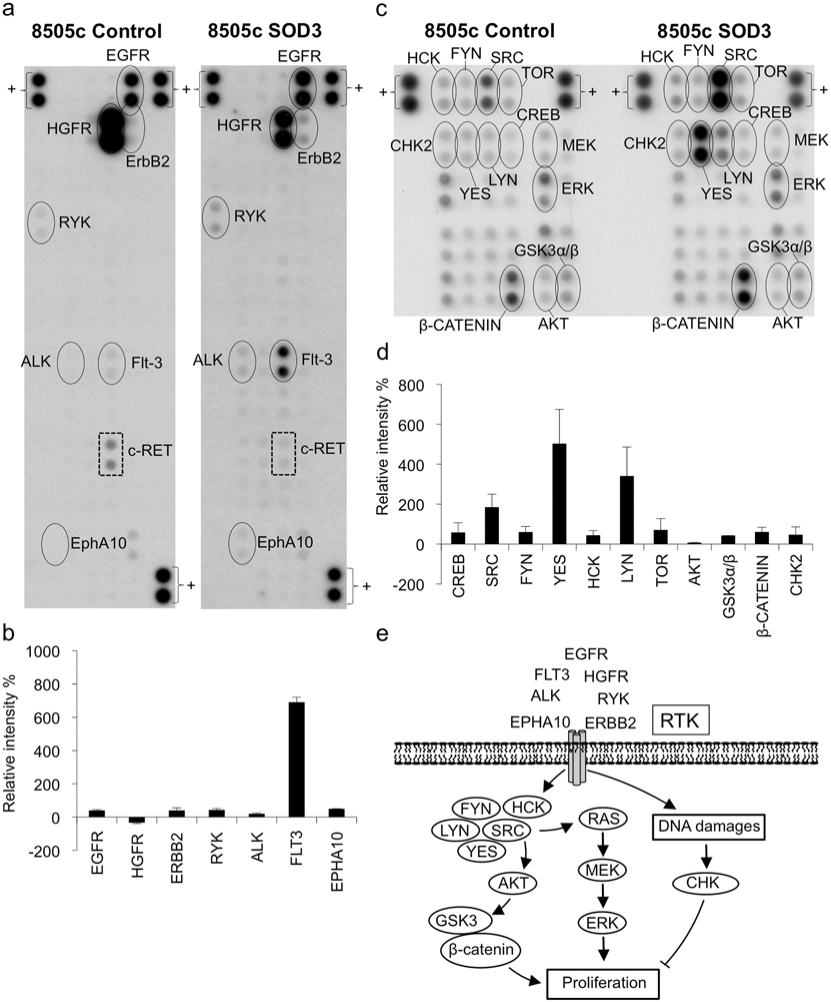
The phosphorylation status of receptor tyrosine kinases and cellular kinases in control plasmid and *SOD3* transfected cells. (a,b) A receptor tyrosine kinase phosho-array showed increased levels of phosphorylation of FLT-3, EGFR, ERBB2, RYK, ALK, and EPHA10 in the 8505c SOD3 cells while HGFR activation was downregulated. Note, c-RET is not expressed in 8505c cells and therefore the signal represents unspecific labeling (S1 Fig.). (c,d) Kinase phosphoarray for cytosolic non-receptor kinases and other signaling molecules. *SOD3* over-expression increased the activation of CREB, TOR, AKT, GSK3α/β, CHK2, and SCR family members SRC, FYN, YES, HCK, and LYN. Additionally, SOD3 induced β-catenin production. (e) A schematic representation of the main pathways that are affected by robust SOD3 over-expression.

SOD3 is a secreted molecule that interacts reversibly with the cell membrane-associated heparan sulfate proteoglycan molecules, such as collagen [15] and fibulin-5 [16]. To study the effect of high SOD3 expression on the phosphorylation of cell membrane-associated growth-related molecules we analyzed its effect on the activation of receptor tyrosine kinases. The dot intensity analysis of the phospho tyrosine kinase receptor array suggested an increased level of phosphorylation of epithelial growth factor receptor (EGFR), receptor like tyrosine kinase (RYK), anaplastic lymphoma kinase receptor (ALK), fms tyrosine kinase 3 receptor (FLT-3), ephrin A10 receptor (EPHA10), and v-erb-b2 avian erythroblastic leukemia viral oncogene homolog 2 (ERBB2, HER2) (Fig. 2a and 2b), which are commonly activated in cancer cells [17,18,19,20,21]. In contrast, the level of phosphorylation of HGFR was reduced in the SOD3-positive cells (Fig. 2a and 2b).

### High SOD3 over-expression affected the activation of the AKT/GSK/β-catenin pathway, ERK1/2 pathway, and CHK2 kinase signal transduction pathways

The tyrosine kinase receptors transmit extracellular stimuli to membrane-associated non-receptor tyrosine kinases and small GTPases [22]. To investigate the effect of SOD3 on the activation of intracellular signaling pathways, we evaluated the phosphorylation status of non-receptor tyrosine kinases using a phospho-kinase array. Based on the dot-intensity analysis of an array representing a selection of RTK downstream kinases, robust SOD3 expression induced the increased phosphorylation of SRC (Tyr419), LYN (Tyr397), YES (Tyr426), HCK (Tyr394), and FYN (Tyr420) (Fig. 2c and 2d). SRC kinases are well-characterized proto-oncogenes that mediate the signaling between receptor tyrosine kinases and RAS [23], thereby inducing the activation of the mitogenic pathway, which leads to cellular proliferation. In addition, SRC activation induces the activation of the AKT-GSK3-β-catenin pathway, another major signal transduction pathway that mediates carcinogenic growth. The PI3K/AKT pathway together with the MEK/ERK signaling pathway is a crucial regulator of cell proliferation, survival and invasion. One of the substrates of AKT is the activation of the β-catenin signaling [24]. AKT inactivates the destruction complex by phosphorylating glycogen synthase kinase-3α/β (GSK3α/β) at serines 21 and 9, thereby leading to the accumulation of β-catenin in the cytoplasm [25]. Subsequently, the cytoplasmic β-catenin is translocated into the nucleus, where it interacts with transcription factors to promote cell growth and carcinogenesis [24]. Our results suggest an activation of the AKT-GSK3-β-catenin pathway in SOD3 expressing cells (Fig. 2c, 2d, and 2e). Lastly, consistent with our previous observations [5], the results of the kinase array assay indicated that high SOD3 over-expression led to the increased phosphorylation of CHK2 kinase (Fig. 2c, 2d, and 2e), which is a component of the DNA damage response signal-transduction pathway and is frequently activated due to DNA-strand breaks [26].

**Fig 3:**
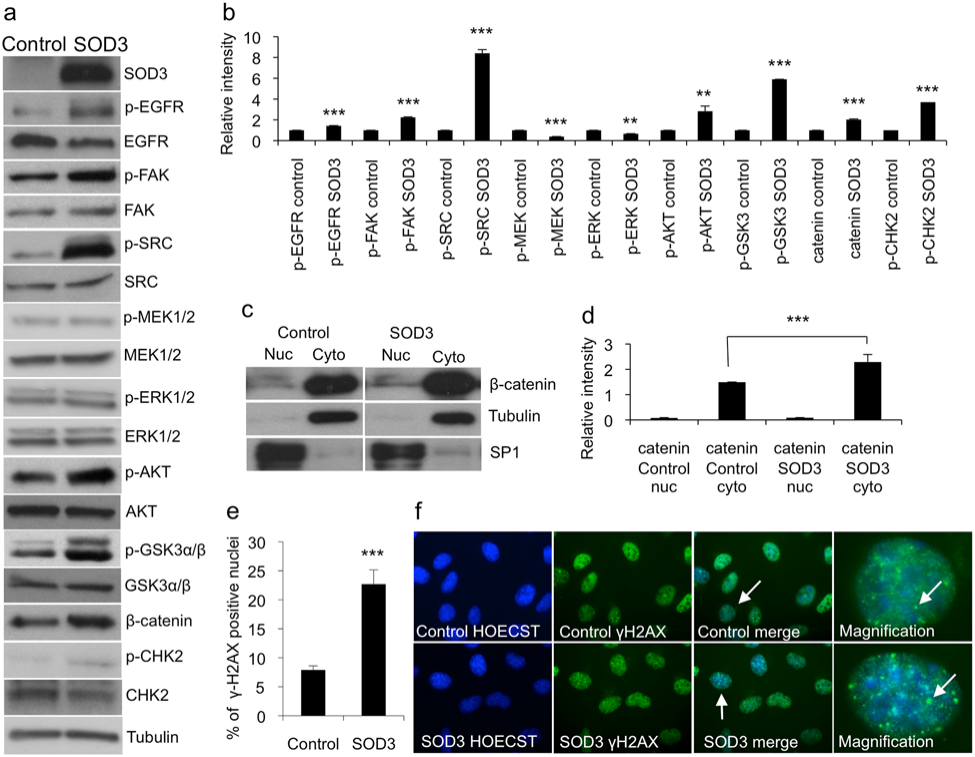
Western blotting and DNA damage response analysis. (a,b) Western blotting analyses supported the protein array data showing the increased activation of RTKs and non-receptor kinases in the *SOD3*-transfected 8505c cells. Note, MEK1/2 and ERK1/2 phosphorylation was moderately downregulated based on the band density analyses. (c,d) Western blotting and the related band intensity analysis for β-catenin nuclear and cytosolic proteins showed significantly (p<0.001) increased β-catenin production in cytosolic compartment. Tubulin was used to normalize cytosolic proteins and SP1 was used to normalize nuclear proteins. (e,f) γH2AX staining for 8505c cells suggested significantly (p<0.001) increased histone H2AX phosphorylation, suggesting DNA damages in 8505c SOD3 cells, which supported the increased CHK2 phosphorylation observed in protoarray and in the Western blotting. The white arrows in the control and the SOD3 merge panels indicate cells that are magnified to detect the nuclear foci. The white arrows in the control and the SOD3 magnification images indicate the nuclear foci. Data are expressed as mean ±SD. The p-values (* = p<0.05, ** = p<0.01, *** = p<0.001).

To confirm the SOD3-mediated pathway activation indicated by the results of the protein array assay and to exclude the artifacts caused by nonspecific antibody binding (Fig. 2a and S1 Fig.), we performed Western blotting analysis of SOD3 over-expressing cells and control cells (Fig. 3a and 3b). The analysis confirmed that a robust SOD3 over-expression increased the level of phophorylation of EGFR, FAK, and SRC indicating that it activated cell membrane-associated RTKs and non-receptor tyrosine kinases. Notably, there was no activation of the downstream kinases MEK1/2 and ERK1/2 but rather a modest decrease in the level of their phosphorylation (Fig. 3a and 3b), which was consistent with the reduced levels of cell proliferation (Fig. 1d, 1e, and 1f) and invasive growth (Fig. 1g, 1e, 1h, and 1i) suggesting the existence of a regulatory step in signal transfer between the SRC and MAP kinases.

Our Western blotting data showed the robust activation of the AKT (Thr308) signaling protein in SOD3 over-expressing cells, consistent with our previous observations [8], which led to the phosphorylation of its substrate GSK3α/β at Ser21/9 and the accumulation of β-catenin (Fig. 3a and 3b). Interestingly, the increased amount of cytosolic β-catenin was not accompanied by an increase in the amount of the nuclear fraction (Fig. 3c and 3d), again suggesting the existence of an inhibitory regulation occurring following strong SOD3 over-expression. Lastly, the Western blotting analysis confirmed the increased activation of CHK2 kinase (Fig. 3a and 3b) upon SOD3 over-expression, which was accompanied by increased H2AX histone phosphorylation (γH2AX) (Fig. 3e and 3f).

### Robust SOD3 over-expression had a significant impact on proliferation, migration, and invasion related gene expression

To gain a global view of the signaling pathways engaged by SOD3 to cause the reduced growth signaling and consequent reduced rate of cell proliferation, we analyzed the transcriptional alterations that occurred upon SOD3 over-expression by performing a microarray analysis of control plasmid and *SOD3* transgene transfected 8505c cells (S2 and S3 Tables).

Analysis of our microarray data revealed that approximately 32% of the SOD3-mediated downregulated and upregulated genes were growth promoters or suppressors (45 of 136 and 20 of 61, respectively), emphasizing the connection between SOD3 and the growth-regulatory genes (Tables 1 and 2). Microarray Gene Ontology (GO) analysis indicated that of the 67 genes involved in the regulation of cell proliferation (GO:0042127), the expression of 24 genes was upregulated and that of 43 genes was downregulated (S4 Table). Of the 33 cell proliferation-related genes (GO:0008283), the expression of 17 genes was upregulated and that of 16 genes was downregulated. In the group of genes involved in the regulation of cell migration (GO:0030334), the expression of 12 of the 27 genes was upregulated, whereas that of the remaining 15 genes was downregulated. Consistent with this result, of the 38 genes in the cell migration group (GO:0016477), the expression of 13 genes was upregulated and that of 25 genes was downregulated. Of the 18 genes in the positive regulation of locomotion group (GO:0040017), the expression of 8 genes was upregulated and that of 10 genes was downregulated. The regulation of cell motility group (GO:2000145) had 25 genes, of which the expression of 11 was upregulated and that of 14 genes was downregulated. Finally, the cell motility group of genes (GO:0048870) included 29 genes, of which 9 showed upregulated expression and 20 showed downregulated expression in the SOD3 over-expressing cells. Thus, the microarray-based functionality analysis indicated that robust SOD3 over-expression mostly downregulated the expression of genes involved in growth and migration.

**Table 1:** Growth-associated genes showing SOD3-mediated downregulated expression.

Growth promotors downregulated by SOD3		
Gene	Definition	Reference
AKR1C3	Aldo-keto reductase family 1, member C3	NM_003739.4
Ang	Angiogenin	NM_001097577.1
ARHGEF16	Rho guanine exchange factor (GEF) 16	NM_014448.2
CCNA1	Cyclin A1	NM_003914.2
CSF2	Colony stimulating factor 2 (granulocyte-macrophage)	NM_001781.1
ELF3	E74-like factor 3	NM_004433.3
DDIT4/REDD1	DNA-damage-inducible transcript 4	NM_019058.2
ERBB3	v-erb-b2 erythroblastic leukemia viral oncogene homolog 3	NM_001982.2
FOXO1	Forkhead box O1	NM_002015.3
GCHFR	GTP cyclohydrolase I feedback regulator	NM_005258.2
HAS3	Hyaluronan synthase 3	NM_005329.2
IL1β	Interleukine 1β	NM_000576.2
LTB	Lymphotoxin beta	NM_002341.1
MAP3K8	Mitogen-activated protein kinase kinase kinase 8	NM_005204.2
MSLN	Mesothelin	NM_013404.3
NUAK2	SNF1-like kinase, 2	NM_030952.1
NOX5	NADPH oxidase 5	NM_024505.2
PDGFRL	Platelet-derived growth factor receptor-like	NM_006207.1
RGL1	Ral guanine nucleotide dissociation stimulator-like 1	NM_015149.3
PLAT	Plasminogen activator, tissue	NM_000930.2
SCD	Stearoyl-CoA desaturase	NM_005063.4
PRSS7	Protease serine 7 (enterokinase)	NM_002772.1
RIPK4	Receptor-interacting serine-threonine kinase 4	NM_020639.2
RRAD	Ras-related associated with diabetes	NM_004165.1
VEGFA	Vascular endothelial growth factor A	NM_001025367.1
VCAM1	Vascular cell adhesion molecule 1, CD106	NM_001078.2
WNT7A	Wingless-type MMTV integration site family, member 7A	NM_004625.3

Growth suppressors downregulated by SOD3		
Gene	Definition	Reference
BIRC3	Baculoviral IAP repeat-containing 3	NM_001165.3
BNIP3L	BCL2/adenovirus E1B 19kDa interacting protein 3-like	NM_014448.2
CCNG2	Cyclin G2	NM_004354.1
EFNA1	Ephrin-A1	NM_004428.2
HRASLS3	HRAS-like suppressor 3	NM_007069.2
JUP	Plakoglobin/γ-catenin	NM_002230.1
KLF9	Kruppel-like factor 9	NM_001206.2
LCN2	Lipocalin 2	NM_005564.3
MTSS1	Metastasis suppressor 1	NM_014751.2
NDRG1	N-myc downstream regulated gene 1	NM_005204.2
PCDH20	Protocadherin 20	NM_022843.2
RASAL1	RAS protein activator like 1 (GAP1 like)	NM_004658.1
RASSF2	Ras association (RalGDS/AF-6) domain family 2	NM_170774.1
RASSF4	Ras association (RalGDS/AF-6) domain family member 4	NM_032023.3
STAT1	Signal transducer and activator of transcription 1	NM_007315.2
STAT2	Signal transducer and activator of transcription 2	NM_005419.2
YPEL3	Yippee-like 3	NM_031477.4
VANGL2	Vang-like 2/Van gogh	NM_020335.1

**Table 2:** Growth-associated genes showing SOD3-mediated upregulated expression.

Growth promotors upregulated by SOD3		
Gene	Definition	Reference
ABCE1	ATP-binding cassette, sub-family E,1	NM_001040876.1
CCND2	Cyclin D2	NM_018404.2
CYR61	Cysteine-rich, angiogenic inducer, 61	NM_001554.3
CTPS	CTP synthase	NM_001905.1
EEF1A2	Elongation factor 1 alpha 2	NM_001958.2
ID1	Inhibitor of DNA binding 1	NM_181353.1
ID3	Inhibitor of DNA binding 3	NM_002167.2
NMU	Neuromedin U	NM_006681.1
PLAC8	Placenta-specific 8	NM_016619.1

Growth suppressors upregulated by SOD3		
Gene	Definition	Reference
ARHGDIB	Rho GDP dissociation inhibitor (GDI) beta	NM_001175.4
ADAP2	ArfGAP with dual PH domains 2	NM_018404.2
DKK1	Dickkopf homolog 1	NM_012242.2
EPHB1	EPH receptor B1	NM_004441.3
GSTM1	Glutathione S-transferase M1	NM_000561.2
GSTM2	Glutathione S-transferase M2	NM_000848.2
HMOX1	Heme oxygenase (decycling) 1	NM_002133.1
PLK2	Polo-like kinase 2 (Drosophila)	NM_006622.2
RGS4	Regulator of G-protein signalling 4	NM_005613.3
THBS1	Thrombospondin 1	NM_003246.2

To validate the microarray data we selected 18 genes involved in growth, migration, and invasion (S2 Fig.) and confirmed using real-time RT-PCR the SOD3-mediated alteration of gene expression. To cluster the genes involved in signal transduction pathways, we applied the categories of the Kyoto Encyclopedia of Genes and Genomes (KEGG) finding 254 genes related to mitogen activated protein kinase (MAPK) signal transduction among the SOD3-regulated transcripts (Table 3). Accordingly, the expression of 312 genes related to oncogenic pathways was affected upon *SOD3* over-expression. Consistent with the results of the KEGG analysis, the stimulated growth-related signaling cascades in SOD3 over-expressing cells that were identified by searching the Gene Ontology project (GO) indicated that cell proliferation and mitogen regulated pathways play a central role in the response to SOD3 over-expression (Table 3). Finally, Gene Ontology (GO) analysis of the microarray data revealed the SOD3-mediated increased expression of the *WWTR1* and *SNAI2* genes, which are responsible for β-catenin cytoplasmic arrest [26] and binding [27], respectively, in addition to the SOD3-mediated decreased expression of *AXIN2*, which drives the degradation of cytoplasmic β-catenin [28] (Table 3). These data supported the results of the protein array and Western blotting analyses, which showed an increased level of β-catenin protein in the cytoplasm of SOD3 over-expressing cells (Fig. 3c and 3d).

**Table 3:** The results of the KEGG, GO, and GOrilla functional analysis.

KEGG signaling pathway analysis			
*Pathway*	*p-value*	*Genes*	*Pathway description*
4010	0.02	254	MAPK signaling pathway
5200	0.023	312	Pathways in cancer
5222	0.026	78	Small lung cancer
5215	0.027	83	Prostate cancer
5219	0.029	39	Bladder cancer
4012	0.047	83	ErbB signaling pathway
5210	0.048	81	Colorectal cancer

GO pathway analysis			
*Pathway*	*p-value*	*Genes*	*Pathway description*
GO:0001935	0.018	33	Endothelial cell proliferation
GO:0001936	0.018	27	Regulation of endothelial cell proliferation
GO:0001937	0.018	17	Negative regulation of endothelial cell proliferation
GO:0001938	0.023	11	Positive regulation of endothelial cell proliferation
GO:0000083	0.034	5	Regulation of transcription of G1/S-phase
GO:0001558	0.036	128	Regulation of cell growth
GO:0000279	0.044	308	M phase
GO:0000188	0.046	18	Inactivation of MAPK pathway

Negative regulation of catenin import into nucleus SOD3 vs. Control				
*GOrilla term*	*p-value*	*Gene*	*Function*	*Fold change*
GO:0035414	7.80E-04	WWTR1	β-catenin cytoplasmic arrest	1.91
		SNAI2	β-catenin cytoplasmic binding	1.77
		AXIN2	β-catenin cytoplasmic degradation	-1.62

The data indicated that SOD3 played a role in the regulation of the expression of genes involved in cell-proliferative pathways.

### Robust SOD3 over-expression modified the expression of the small GTPase regulatory *GEF*, *GAP* and *GDI* mRNAs

**Fig 4:**
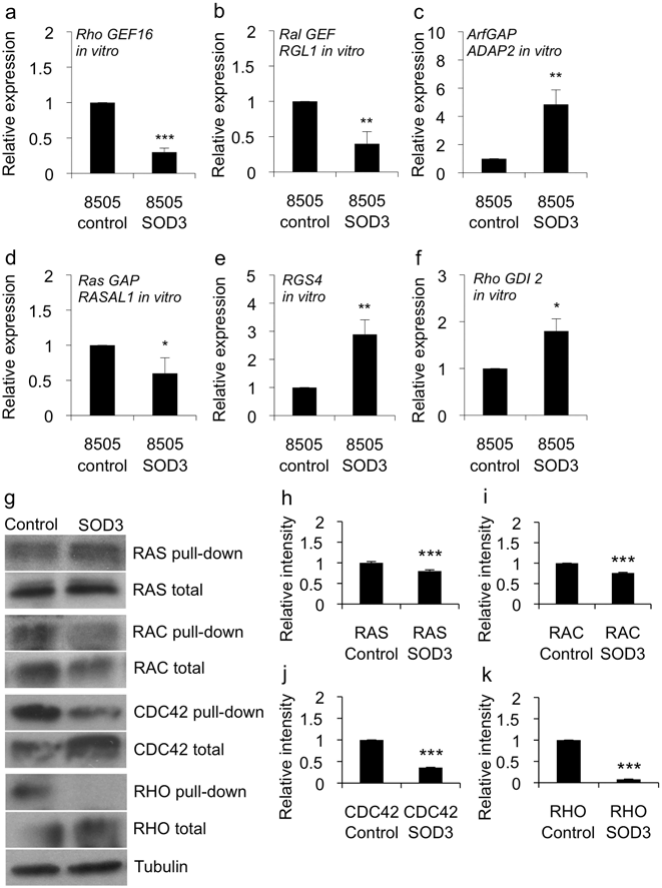
SOD3 regulated the expression of small GTPase regulatory genes and affected the activation of small GTPases. (a-f) Quantitative RT-PCR for GTPase superfamily regulatory genes. The panels show the expression levels of the regulatory genes *in vitro* cultured cells containing high-level *SOD3* mRNA and reduced growth abilities. (g-k) Pull-down assay for small GTPase proteins RAS, RAC, CDC42, and RHO. The assay suggested significantly (p<0.001) reduced GTP loading to each tested GTPase therefore being in line with reduced cell proliferation, migration, and growth related signal transduction. Data are expressed as mean ±SD. The p-values (* = p<0.05, ** = p<0.01, *** = p<0.001).

Interestingly, our microarray data revealed changes in the levels of expression of guanine nucleotide exchange factors (GEFs), GTPase activating proteins (GAPs), and a Rho guanine nucleotide dissociation inhibitor (GDI) that were affected by SOD3 over-expression (Fig. 4a, 4b, 4c, 4e, and 4f). GEFs are involved in the exchange of GDP for GTP, which activates members of the small G-protein superfamily of GTPases, whereas GAPs terminate the active state by inducing GTP hydrolysis [29,30]. GDIs in turn inhibit the dissociation of GDP from small G-protein GTPases thereby maintaining these molecules in their inactive form [31]. Robust *in vitro* SOD3 expression downregulated the level of transcription of *Rho guanine exchange factor 16* (*RHO GEF16*), *Ral guanine nucleotide dissociation stimulator-like 1* (*RGL1*), and *Ras protein activator like 1 (GAP1 like)* (*RASAL1*) genes. Among the genes with upregulated mRNA expression in the SOD3 over-expressing cells, the most relevant GTPase-associated genes were *RHO GDP dissociation inhibitor beta* (*RHO GDI2*), *ArfGAP with dual PH domains 2* (*ADAP2*), and *Regulator of G-protein signaling 4* (*RGS4*), the latter one having also GAP activity (Fig. 4a, 4b, 4c, 4d, 4e, and 4f).

To link these data to the activation of small G proteins, we performed pull-down assays to evaluate the status of the small GTPase RAS, RAC, CDC42, and RHO, which showed a reduced level of small GTPase activation in the *SOD3* over-expressing cells (Fig. 4g, 4h, 4i, 4j, and 4k) supporting the observed decreased activation of the MEK1/2 and ERK1/2 mitogen activated signaling pathway and the decreased rate of cellular proliferation in these cells. These data hence indicated the existence of a major SOD3-related regulatory mechanism that affected the activation of the membrane-associated small GTPase molecule.

### SOD3 over-expression regulates small GTPase regulatory gene mRNA production in a dose dependent manner

**Fig 5:**
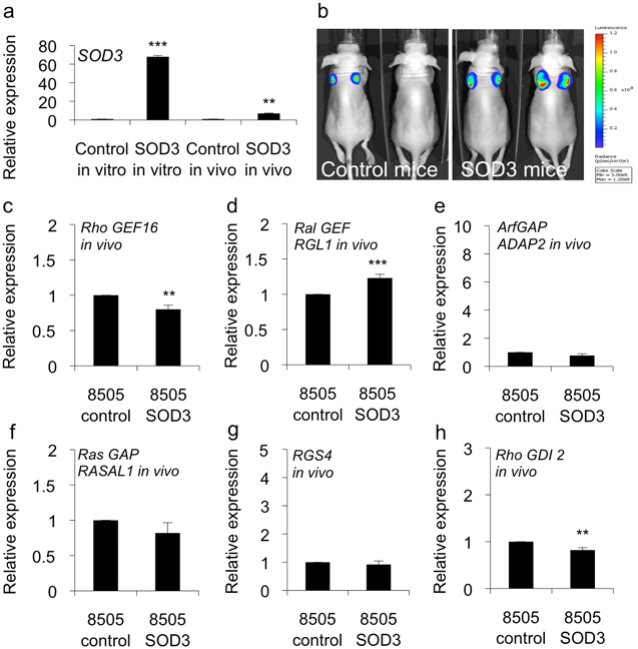
*In vivo* transplantation of 8505c SOD3 cells results in tumorigenesis. (a) There was a robust over-production of *SOD3* mRNA in 8505c SOD3 stable cells while the tumors derived from 8505c SOD3 stable cells contained moderately increased *SOD3* mRNA consistent with our previously published data. (b) Bioluminescence imaging showing the increased tumor size in 8505c SOD3 transplanted mice as compared to control cell transplanted animals. (c-h) *GEF*, *GAP*, and *GDI* mRNA expression *in vivo*. The mice showing a moderately increased SOD3 expression in transplanted 8505c cells had reversed small GTPase regulatory gene expression as compared to *in vitro* situation. Data are expressed as mean ±SD. The p-values (* = p<0.05, ** = p<0.01, *** = p<0.001).

We previously demonstrated that the effect of SOD3 on the expression of growth-related genes correlated with the level of expression of this enzyme *in vitro* and *in vivo*. A robust SOD3 expression caused growth arrest, whereas a moderately increased SOD3 production supported cell survival, growth and tumorigenesis [5,6,8,32]. In the current study, we evaluated mice that had been previously transplanted with 8505c cells transfected with the *luciferase* expression control vector or *SOD3* expression vector [5] to study the effect of low-dose growth promoting SOD3 synthesis levels on the expression of GEFs, GAPs, and GDIs (Fig. 5a and 5b). While the *in vitro* expression of GTPase regulatory genes favored the reduced growth rate and the inactivation of small GTPases (Fig. 4a, 4b, 4c, 4d, 4e, and 4f), the level of mRNA expression of *GEF*, *GAP*, and *GDI* genes *in vivo* isolated tumors was reversed (Fig. 5c, 5d, 5e, 5f, 5g, and 5h) supporting the observation of low SOD3 level induced increased rate of tumor growth (Fig. 5b) [5]. Although a significantly decreased expression of the *RHO GEF16* and *RAL GEF RGL1* genes was observed *in vitro* in the 8505c SOD3 over-expressing cells (Fig. 4a and 4b), the level of expression of these genes was increased in tumors that showed a moderate expression level of SOD3 *mRNA* (Fig. 5c and 5d). Similarly, the significantly increased expression of the GTPase activator proteins *Arf GAP ADAP2* and *RGS4* was correlated with a high level of *SOD3* expression *in vitro* (Fig. 4c and 4e) and was markedly decreased in the *in vivo* tumors (Fig. 5e and 5g). Finally, the expression of *RHO GDI 2*, of which increased mRNA levels were found *in vitro* (Fig. 4f), was downregulated *in vivo* (Fig. 5h).

**Fig 6:**
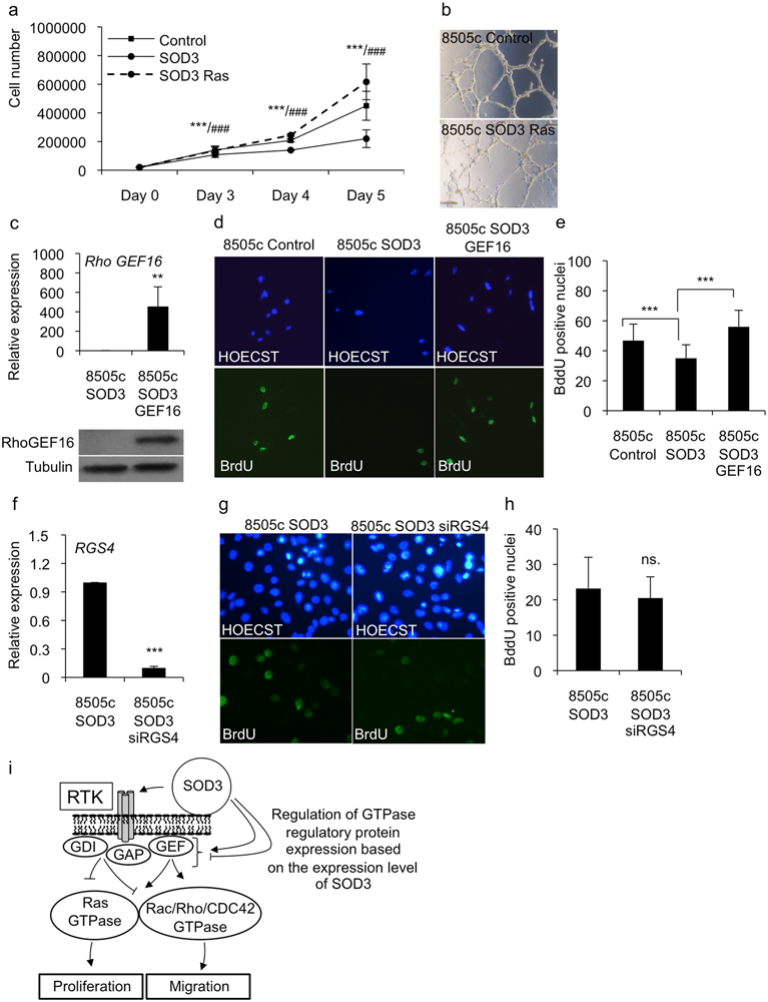
Reversed growth characteristics by *H-RasV12* and *RHO GEF16* over-expression in *SOD3* transfected cells. (a,b) The 8505c SOD3 cells were stable transfected with *H-RasV12* oncogene and analyzed by growth curve and 3-D invasion analysis. Both analysis suggested marked increase in the growth of SOD3 cells after *H-RasV12* transfection. (c) The 8505c SOD3 cells were stably transfected with *RHO GEF16*. The real time RT-PCR suggested significant (p<0.01) increase in *RHO GEF16* mRNA production that was confirmed by Western blotting (lower panel). (d,e) The BrdU DNA incorporation analysis showed significantly (p<0.001) increased nuclear proliferation after *RHO GEF16* over-expression as compared to 8505c SOD3 cells. (f) The RNAi of *RGS4* in 8505c SOD3 cells showed significant downregulation of *RGS4* mRNA production. (g,h) However, the BrdU DNA incorporation analysis failed to show differences between 8505c control and 8505c SOD3/siRGS4 cells. (i) A schematic drawing hypothesizing the SOD3 action in the cells. SOD3 over-expression activates cell membrane RTKs and membrane associated non-receptor tyrosine kinases. Depending on the expression level of *SOD3* the proliferative and migratory signal is either halted or promoted at the level of small GTPase regulatory genes and small GTPases.

To dissect the effects of SOD3 on the expression of the small GTPase regulatory genes, we investigated the functional effects of *RAS* and *RHO GEF16* over-expression in *SOD3* transfected cells. Our data demonstrated that the stable expression of the *H-RasV12* oncogene in 8505c *SOD*3 cells reverted the SOD3-mediated growth inhibitory effect, confirming the negative regulation of RAS by SOD3 (Fig. 4g and 4h) and suggesting a role for RAS as a mediator of SOD3 function, as previously described (Fig. 6a) [6]. Analysis of the invasive capability as determined using 3D Matrigel assay confirmed the ability of *H-RasV12* to revert the growth inhibition of SOD3 cells to the levels of 8505c control cells (Fig. 6b). Similarly to the effect of *H-RasV12* expression, the over-expression of the *RHO GEF16* gene in 8505c SOD3 cells caused an increased level of cell proliferation compared with that of the 8505c control and 8505c SOD3 cells (Fig. 6c, 6d, and 6e). The data suggests that SOD3-mediated inhibition of *RHO GEF16* expression is an important step in the SOD3-mediated regulation of cell growth, which supports previous observations concerning the oncogenic character of *RHO GEF16* [33]. In contrast, RNAi of *RGS4* expression had no effect on the growth potential of SOD3 expressing cells, suggesting that modulation of a single gene encoding a protein with GAP activity was insufficient to inhibit the effects of SOD3 over-expression (Fig. 6f, 6g, and 6h).

The 8505c control, 8505c SOD3, 8505c SOD3 Ras, and in 8505c SOD3 GEF16 cells were stained with dihydroethidium (DHE) and with MitoTracker RED CMX. DHE staining of live cells suggested no difference between 8505c control and 8505c SOD3 cells, a modest increase in cytoplasmic staining in 8505c SOD3 Ras cells as compared to control cells and a marked increased stainining in 8505c SOD3 GEF16 cells (S3 Fig.). MitoTracker RED CMX staining suggested similar mitochondrial staining in the all cells (S3 Fig.).

In summary, our data revealed a dose-dependent effect of SOD3 on the activity of small GTPase regulatory genes. These data clarified the correlation between high SOD3 expression levels and reduced cell proliferation, colony formation, and cell migration. These effects are regulated by a large set of genes that appear to converge on the small GTPase regulatory genes (Fig. 6i), β-catenin, and growth promoting/suppressing genes.

## Discussion

Reactive oxygen species O_2_^.-^ and H_2_O_2_, which were originally thought to be harmful free radicals, are important second messengers in cellular signaling. The primary reactive oxygen species O_2_^.-^ is a precursor to a number of secondary radicals, such as H_2_O_2_, which can induce DNA damage, lipid peroxidation, and protein oxidation [3]. Noteworthy, at a low physiological concentration, H_2_O_2_ functions as a growth stimulator that increases the rate of cell proliferation whereas at high concentrations, H_2_O_2_ has toxic effects on cellular functions [3]. Superoxide dismutases (SODs) have been traditionally classified as antioxidative enzymes that remove harmful O_2_^.-^ radicals, producing more stable, although still reactive, H_2_O_2_ molecules. Thus SODs regulate the availability of O_2_^.-^ and H_2_O_2_ and should be considered intermediary molecules between oxidative free radical-forming enzymes and antioxidative free radical removing enzymes.

A recent report demonstrated that SOD3 acted as a growth regulator suggesting its ability to affect RAS activation and to stimulate mitogenic growth signaling [6]. However, the mechanism for SOD3—derived RAS activation is poorly characterized. In the present work we analyzed the effect of high SOD3 expression on the phosphorylation status of the cell membrane-associated RTKs and the cytoplasmic non-receptor kinases as well as on the transcriptome to achieve a global view of the transcriptional changes occurring during SOD3 expression. Consistent with the results of previous studies, our functional analysis showed that robust SOD3 expression reduced cellular growth (Fig. 1). Interestingly, high-level SOD3 expression markedly increased the activation of the cell membrane-associated RTKs and the downstream signaling molecules, such as the SRC protein family members SRC, FYN, YES, HCK, and LYN (Fig. 2) all of which function upstream of the small GTPase proteins, such as RAS [34]. More interestingly, the greatly increased level of SRC family signaling in 8505c SOD3 cells did not increase the activation status of small GTPase proteins as compared to control cells (Fig. 4); in contrast, the levels of activation of these proteins in the former cells were moderately decreased thus confirming our earlier observations that SOD3 regulates mitogen signaling at the level of RAS GTPase [6]. The neutralization of the activity of the mitogen signal transduction pathway (Fig. 3a and 3b) was associated with the modification of β-catenin signaling, suggesting that the reduced nuclear localization of β-catenin in SOD3 over-expressing cells (Fig. 3a, 3b, 3c, and 3d) and the simultaneous DDR activation (Fig. 3a, 3b, 3e, and 3f) enhanced the resultant decrease in the rate of cell proliferation observed in the cell proliferation and cell migration experiments (Fig. 1).

The involvement of SOD3 in the growth-regulated signal transduction was confirmed by the microarray screening combined with KEGG, GO, and GOrilla functional analyses (Table 3). The microarray results further demonstrated the SOD3–driven regulation of the genes responsible for the activation of small GTPases. Most importantly, the screening indicated that SOD3 regulated the expression of six GEFs, GAPs, and GDIs (Figs. 4 and 5) that together with the downregulated growth promoters, and the upregulated growth suppressors (Tables 1, 2, and 3) could have caused decreased mitogen signaling despite the presence of a strong upstream mitogen stimulus. Noteworthy, the simultaneous expression of SOD3 and RAS or SOD3 and RHO GEF16 increased the rate of cellular growth to the levels of the control cells strengthening the hypothesis that RAS and RHO GEF16 are mediators of the SOD3 effect on growth and migration (Fig. 6).

Small GTPases play pivotal roles in the regulation of the cytoskeletal rearrangements and cell motility, but are also involved in cell proliferation, transformation and differentiation, and participate in many steps for carcinogenesis initiation and the progression of cancers. Once bound to the cell membrane, the GTPase superfamily members interact with the upstream stimulators, such as SRC family proteins, and the cytoplasmic molecules that tightly are regulating their activity [34]. The activation of small GTPases requires GDP-GTP exchange, which is catalyzed by various guanine nucleotide exchange factors (GEFs) [35,36]. In contrast, GTPase-activating proteins (GAPs) inactivate small GTPases by stimulating the hydrolysis of GTP [35,36]. Inactive GTPases are sequestered in the cytosol by the guanine nucleotide dissociation inhibitors (GDI) [35,36,37]. Due to their ability to increase the activity of small GTPase superfamily, GEFs are potential oncogenes that affect tumorigenesis, e.g. RAL GEFs have been shown to increase the anchorage independent growth *in vitro* and the transformation activity after their membrane translocation [38].

GAPs activate the intrinsic GTPase activity of Ras superfamily GTPase members, which hydrolyze GTP and return the GTPase to the inactive GDP bound form. Interestingly, GAPs can hydrolyze only the wild-type RAS to terminate its signaling, whereas these molecules have no effect on mutant RAS activity, therefore being less attractive drug targets [39,40]. However, several studies have shown the tumor suppressive properties of GAPs [41] hence suggesting that they play a role in early stages of tumorigenesis.

The third group of regulators is GDIs, which maintain the GTPases in GDP-bound inactive form in the cytosolic compartment and control their GTPase membrane-binding capacity. RHO GDI2 has been shown to be a metastasis suppressor in bladder and breast cancers [42,43]. In line, the level of RHO GDI2 expression was increased during the early phase of breast epithelial hyperplasia and was significantly decreased in transformed metastatic cells. Indeed, it has been shown that RHO GDI2 reduced the extend of cell migration and served as a metastasis inhibitor rather than a cell proliferation suppressor [44].

We have recently demonstrated that SOD3 has a dose-dependent effect on cellular growth *in vitro* and *in vivo* [5]. High-level SOD3 expression decreased the growth rate by inducing apoptosis in p53 responsive cancer cells whereas a moderately upregulated level of SOD3 production increased the proliferation of primary and cancer cells, and promoted cellular immortalization and neoplastic transformation. Noteworthy, the induction of p53 activation and the resultant growth arrest were observed in both in primary cells and in cancer cells containing functional p53 response. Furthermore, the p53 induction occurred at both at low and high *SOD3* expression levels: the response induced by high-level *SOD3* expression was the immediate death of most of the cells within 24 hours, whereas low-level *SOD3* expression first activated a proliferative burst that was followed by DNA strand break stimulated p53-p21 growth arrest and a senescence phase in a fashion similar to that caused by the *H-RasV12* oncogene [5]. However, the growth arrest pathway activation does not solely explain this reduced cellular growth. A number of *in vivo* SOD3 mouse model studies have demonstrated the decreased level of tumorigenesis under both p53-responsive and p53-inactive conditions, supporting our recent *in vitro* data [45,46,47,48] and indicating the existence of additional growth-signaling-related regulatory systems. Therefore, the current data showing decreased *GEF* expression and increased *GAP* and *GDI* production, which paralleled with a reduced activation of small GTPases at high SOD3 levels, together with the observed effects on β-catenin signaling supported the previous observations and may explain how robust SOD3 expression reduces cellular growth. In turn, a moderately increased *SOD3* mRNA expression reversed the *GEF*, *GAP*, and *GDI* synthesis suggesting the regulatory characteristics of SOD3 in controlling the activation the status of small GTPases.

Our current and previous data thus suggest that SOD3 has a dose-dependent effect on growth signaling. A moderate level of SOD3 expression induced proliferative signal transduction through phophorylation of the RTKs and cytosolic non-receptor kinases, leading to RAS and ERK activation. In contrast, high SOD3 levels reduced the rates of cell proliferation and cell migration by affecting the expression of a family of small GPTase regulatory genes that negatively regulate the activity of small GTPases and ERK kinases. These data revealed a new scenario, in which SOD3 acts in a dose-dependent manner to simultaneously activate distinct pathways that have conflicting effects. Because SOD3 itself is a drug target of great interest, the current study might indicate molecules that mediate the effects of SOD3 on cell proliferation and hence are potential druggable targets in the early phase of tumorigenesis.

## Supporting Information

S1 Fig Western blotting analysis showing a false positive signal. RET and phospho-RET Western blotting from 8505c cells showed no RET expression. The bands observed in the p-RET lane are the result of nonspecific labeling.
(TIF)

S2 Fig Quantitative RT-PCR validation for the microarray. (TIF)

S3 Fig Reactive oxygen staining from live cells. (a) The images show Hoechst nuclear staining and DHE ROS staining. DHE staining of the cells shows minor differences in ROS staining in 8505c SOD3 as compared to 8505c control cells. A moderate increase was observed in 8505c SOD3 Ras cells and a marked increased in 8505c SOD3 GEF16 cells. (b) Mitochondrial ROS staining showed no differences between cell lines. Images are shown in grey scale (left side panes) and with red fluorescence (right side panels).
(TIF)

S1 Table Primer sequences used for quantitative RT-PCR. (XLSX)

S2 Table SOD3—derived down-regulation of genes analyzed by microarray. (XLS)

S3 Table SOD3—derived upregulation of genes analyzed by microarray. (XLS)

S4 Table Functional classification of genes using Gene Ontology (GO) analysis. (XLS)
