# Supplementary figures and images for: Extracellular Superoxide Dismutase Regulates the Expression of Small GTPase Regulatory Proteins GEFs, GAPs, and GDI
Source: PLoS One. 2015 Mar 9;10(3):e0121441. doi: 10.1371/journal.pone.0121441 (PMC4353720; doi:10.1371/journal.pone.0121441)

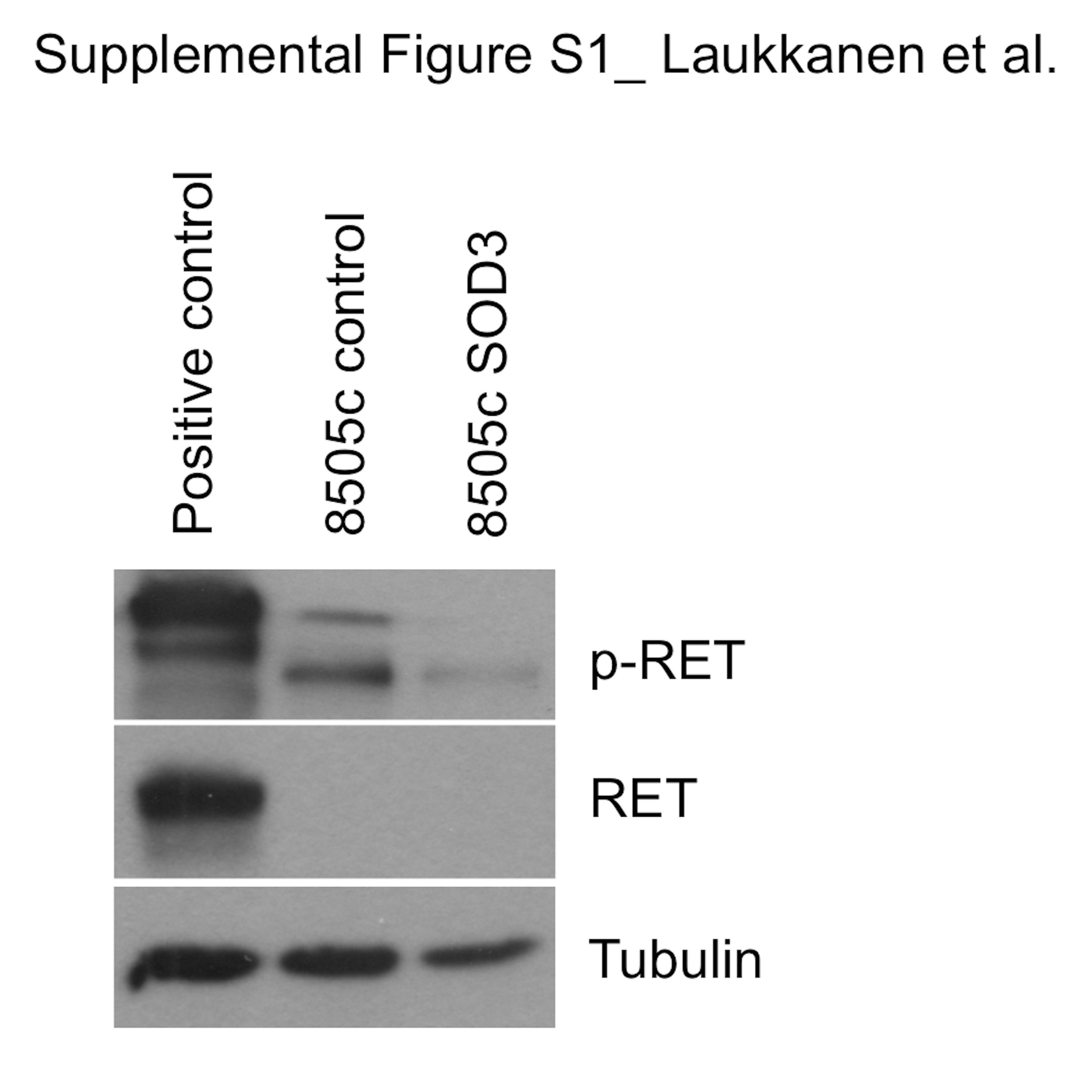

Supplement: S1 Fig — RET and phospho-RET Western blotting from 8505c cells showed no RET expression. The bands observed in the p-RET lane are the result of nonspecific labeling. (TIF) [file pone.0121441.s001.tif]

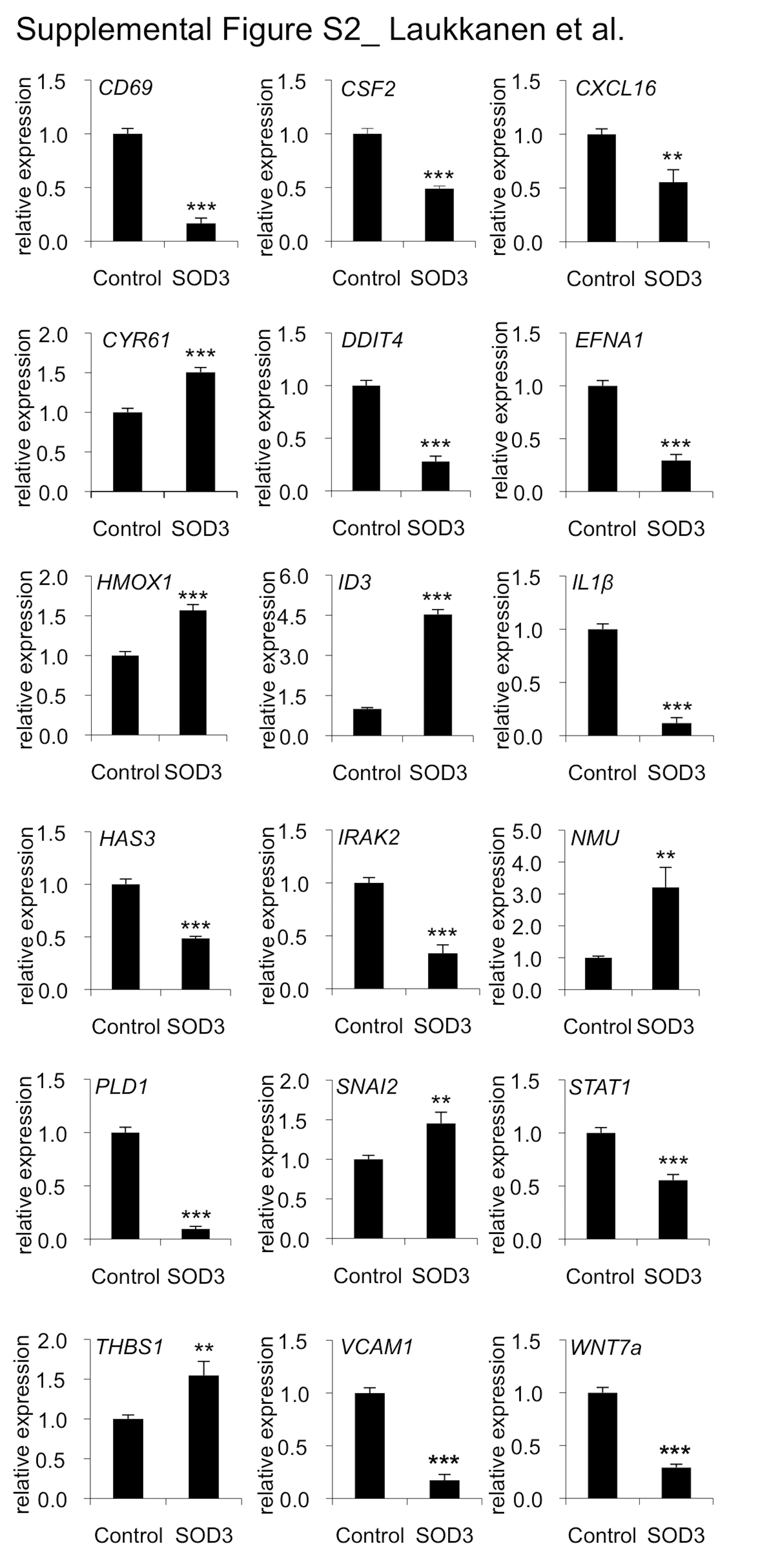

Supplement: S2 Fig — (TIF) [file pone.0121441.s002.tif]

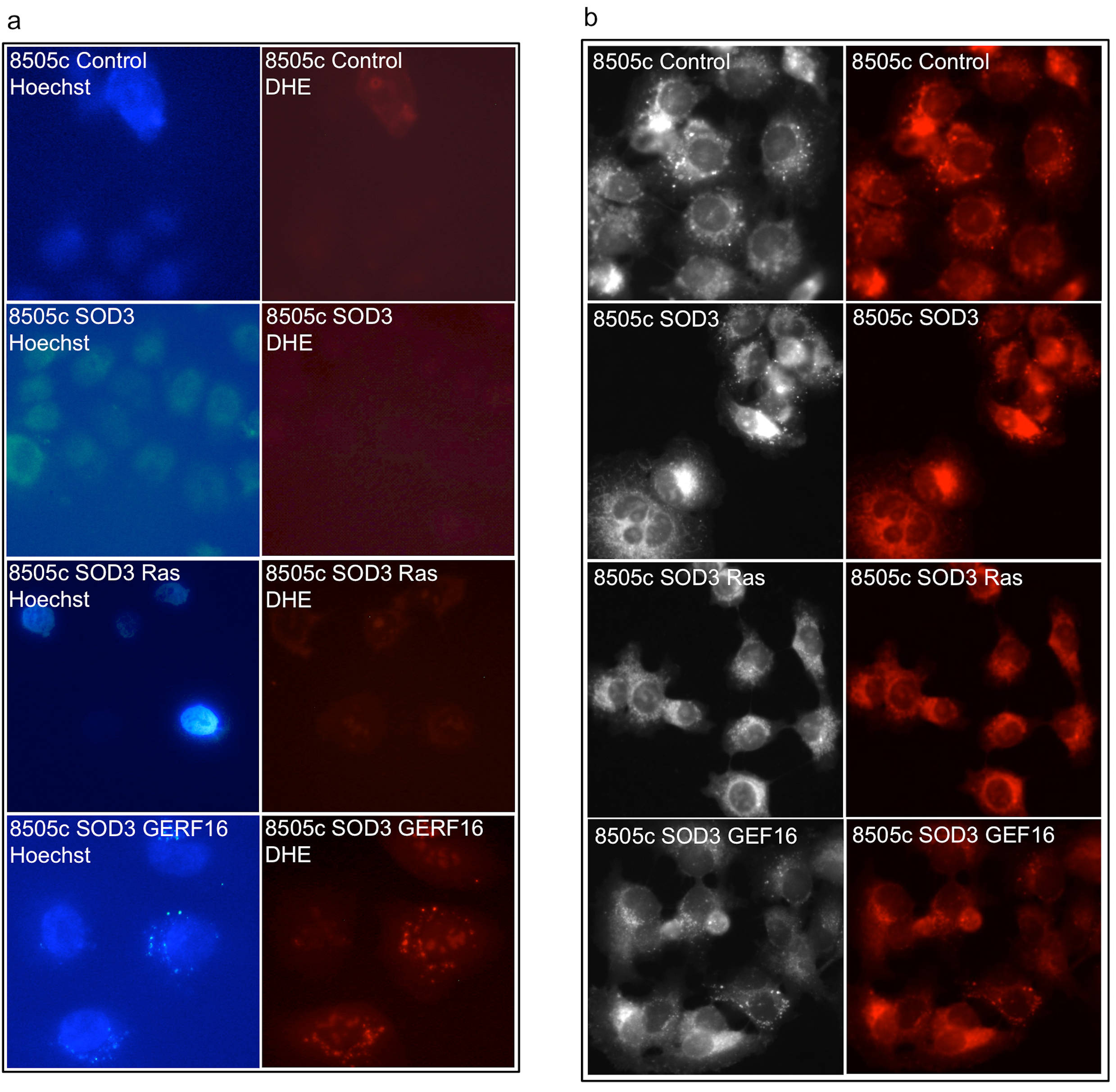

Supplement: S3 Fig — (a) The images show Hoechst nuclear staining and DHE ROS staining. DHE staining of the cells shows minor differences in ROS staining in 8505c SOD3 as compared to 8505c control cells. A moderate increase was observed in 8505c SOD3 Ras cells and a marked increased in 8505c SOD3 GEF16 cells. (b) Mitochondrial ROS staining showed no differences between cell lines. Images are shown in grey scale (left side panes) and with red fluorescence (right side panels). (TIF) [file pone.0121441.s003.tif]
